# A human iPSC line capable of differentiating into functional macrophages expressing ZsGreen: a tool for the study and *in vivo* tracking of therapeutic cells

**DOI:** 10.1098/rstb.2017.0219

**Published:** 2018-05-21

**Authors:** Martha Lopez-Yrigoyen, Antonella Fidanza, Luca Cassetta, Richard A. Axton, A. Helen Taylor, Jose Meseguer-Ripolles, Anestis Tsakiridis, Valerie Wilson, David C. Hay, Jeffrey W. Pollard, Lesley M. Forrester

**Affiliations:** 1 Centre for Regenerative Medicine, Scottish Centre for Regenerative Medicine, University of Edinburgh, 5 Little France Drive, Edinburgh EH16 4UU, UK; 2 Centre for Reproductive Health, The Queen's Medical Research Institute, University of Edinburgh, 47 Little France Crescent, Edinburgh EH16 4TJ, UK

**Keywords:** iPSCs, genome editing, ZsGreen reporter, macrophage

## Abstract

We describe the production of a human induced pluripotent stem cell (iPSC) line, SFCi55-ZsGr, that has been engineered to express the fluorescent reporter gene, ZsGreen, in a constitutive manner. The CAG-driven ZsGreen expression cassette was inserted into the *AAVS1* locus and a high level of expression was observed in undifferentiated iPSCs and in cell lineages derived from all three germ layers including haematopoietic cells, hepatocytes and neurons. We demonstrate efficient production of terminally differentiated macrophages from the SFCi55-ZsGreen iPSC line and show that they are indistinguishable from those generated from their parental SFCi55 iPSC line in terms of gene expression, cell surface marker expression and phagocytic activity. The high level of ZsGreen expression had no effect on the ability of macrophages to be activated to an M(LPS + IFNγ), M(IL10) or M(IL4) phenotype nor on their plasticity, assessed by their ability to switch from one phenotype to another. Thus, targeting of the *AAVS1* locus in iPSCs allows for the production of fully functional, fluorescently tagged human macrophages that can be used for *in vivo* tracking in disease models. The strategy also provides a platform for the introduction of factors that are predicted to modulate and/or stabilize macrophage function.

This article is part of the theme issue ‘Designer human tissue: coming to a lab near you’.

## 1. Introduction

The production of functional mature cell types from induced pluripotent stem cells (iPSCs) is well established as a tool for disease modelling and drug testing and significant progress is being made for their future use in cell therapy [1,2]. Defined protocols have been described that allow the production of relatively pure populations of many terminally differentiated cell types including cardiomyocytes, neurons, hepatocytes, pancreatic beta cells and various cell types of the haematopoietic lineage [3–10]. The unlimited capacity of iPSCs to expand *in vitro* and the advances in genome-editing technology allow them to be genetically manipulated with ease. Gene knockouts can be performed to assess the role of specific genes in healthy or disease states, cell fate can be modulated by genetic programming and genetic tags can be introduced to allow tracking of therapeutic cell populations *in vivo*.

Cells of the monocyte/macrophage lineage have attracted much attention in the cell therapy field as they have been shown to have an anti-fibrotic and pro-regenerative effect in diseases associated with many tissues [11]. For example, in murine disease models, transplantation of bone marrow-derived macrophages mediated the progression and recovery of liver fibrosis [12,13] and improved the disease phenotype of pulmonary alveolar proteinosis [14]. In the first steps to translation, the delivery of human macrophages to an immunocompromised mouse model of liver injury improved liver function and reduced fibrosis [15]. GMP-compliant protocols have been used to derive autologous macrophages from patient's peripheral blood monocytes and these are being used in the first clinical trial of macrophage cell therapy [15,16]. However, it is challenging to derive cells from diseased patients and the time taken to produce a sufficient quantity of therapeutic macrophages severely limits this approach. Thus, the next logical step is to consider an off-the-shelf source of macrophages derived from a source such as iPSCs.

Numerous reports have demonstrated that macrophages can be generated efficiently from human iPSCs and these protocols have been adapted to be GMP-compliant [9,17,18]. It has been suggested that iPSC-derived macrophages are more akin to tissue-resident rather than monocyte-derived macrophages predicting that they might have even more powerful therapeutic properties than patient monocyte-derived cells in some disease settings [18,19]. We demonstrated that macrophages produced *in vitro* from mouse embryonic stem cells (ESCs) were effective in ameliorating fibrosis in a CCl_4_-induced liver injury model *in vivo*, providing the first proof of concept that this strategy is feasible [18]. We also showed that ESC-derived macrophages were more efficient at repopulating the Kupffer cell compartment of mice that had been depleted of macrophages using liposomal clodronate, providing further support for their phenotype being comparable to tissue resident macrophages [18].

It has been notoriously difficult to genetically manipulate primary macrophages, so their production from iPSCs allows the generation of ‘engineered’ macrophages carrying specific gene knockouts, genetic factors that might alter their phenotype or, as described here, a fluorescent label for their tracking *in vivo*. Targeting of transgenes to the *AAVS1* locus of iPSCs and the subsequent differentiation into macrophages *in vitro* solves transgene silencing issues that have been associated with the manipulation of primary macrophages and cells differentiated from pluripotent cells *in vitro* [20–22].

Here, we show that neither the targeting of *AAVS1* locus nor the expression of the ZsGreen reporter affects the production of macrophages from iPSCs. In addition, this genetic manipulation has no effect on macrophage function nor on their ability to be activated into specific phenotypes. iPSC-derived macrophages retain a degree of plasticity and this feature is also unaffected by the genetic manipulation performed in this study. Our data indicate that this powerful platform could be used to study the therapeutic properties of macrophages and the ZsGreen-expressing iPSC-derived macrophages generated in this study could be used to track these therapeutic cells *in vivo*.

## 2. Material and methods

### (a) AAVS1-ZsGreen vector construction

ZsGreen1-polyA sequence was amplified from the pZsGreen1-1 vector (Clontech). CAG promoter was obtained from EcoRV-digested pCAG-IRES-puro plasmid (pCAG-SIP) [23].

**Figure 1.**
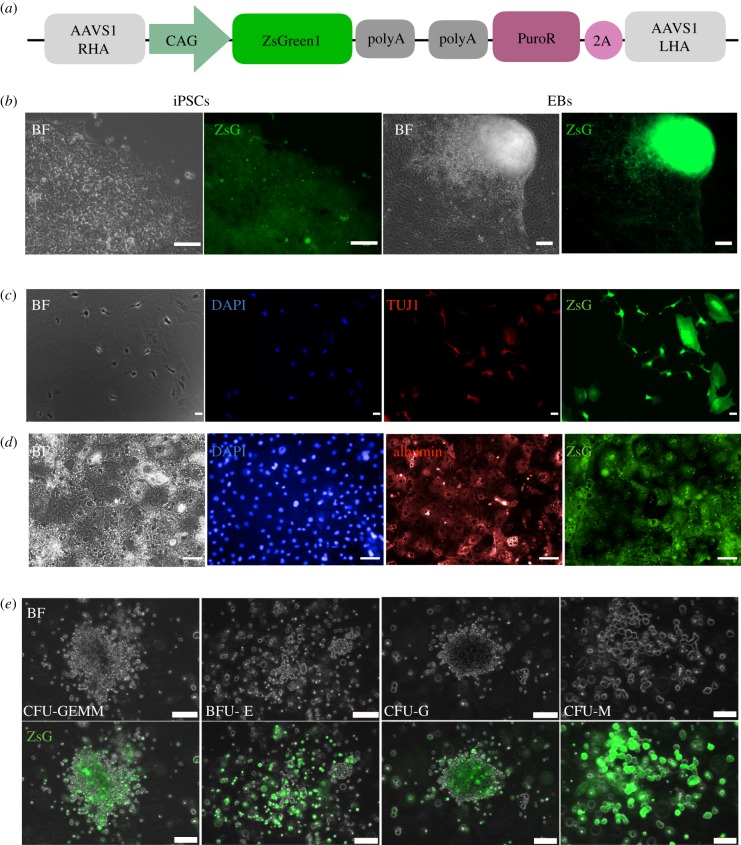
Expression of ZsGreen in undifferentiated and differentiated SFCi55ZsG iPSCs. (*a*) Schematic of targeting vector consisting of the CAG constitutive promoter driving ZeisGreen1 cDNA; the puromycin selectable marker (PuroR), 2A peptide and right (RHA) and left (LHA) homology arms of *AAVS1* locus. (*b*) Bright field (BF) and fluorescent images (ZsG) of SFCi55-ZsG iPSC (left, 40×) and embryoid bodies (EBs) (right, 20×). (*c*) SFCi55-ZsG-derived neurons stained with DAPI and anti-TUJ1 antibody (20×). (*d*) SFCi55-ZsG-derived hepatocytes stained with DAPI and albumin antibody (40×). (*e*) Bright field and fluorescent images of haematopoietic colonies generated in methylcellulose from SFCi55-ZsG (40×). Colony-forming unit granulocyte, erythroid, macrophage, megakaryocyte (CFU-GEMM), burst forming unit erythroid (BFU-E), colony-forming unit, granulocyte, (CFU-G), colony-forming unit, macrophage (CFU-M); (Scale bars, 100 µm).

Both fragments were inserted to the pZDonor-AAVS1 Puromycin vector (PZD0020, Sigma-Aldrich) to generate the CAG-ZsGreen-PolyA cassette (figure 1*a*)

### (b) iPSC maintenance and electroporation

The human iPSC line, SFCi55 was generated in-house [24] and maintained in STEMPRO^®^ hESC SFM prepared by supplementing DMEM/F12 (Gibco) with Glutamax (Gibco), StemPro supplement (Gibco), 1.8% BSA (Gibco), 0.1 mM β-mercaptoethanol (Gibco) and 20 ng ml^−1^ human basic FGF (Invitrogen). Following electroporation [24], cells were plated on wells precoated with CTS™ CELLstart™ substrate (Gibco) in STEMPRO^®^ hESC SFM containing 20 ng ml^−1^ bFGF and 10 μM Rock inhibitor (Merck) and 0.6 μg ml^−1^ puromycin for 10 days. Puromycin-resistant colonies were picked, expanded and genomic DNA was extracted using the MasterPure™ complete DNA and RNA purification kit (Epicentre) according to the manufacturer's instructions. Genomic DNA was PCR screened to identify clones with successful integration into the *AAVS1* locus by homologous recombination.

### (c) Neuronal differentiation

Neuronal differentiation was initiated by first differentiating for 3 days into neuromesodermal progenitors as described [25]. These were subsequently differentiated into motor neurons using a protocol based on previously published culture conditions [26] and stained using an anti-tubulin-β3 (TUBB3) antibody (1 : 1000) (Biolegend).

### (d) Hepatocyte differentiation

Adapted from previous reports [6], iPSCs were maintained on pre-coated laminin 521 (Biolaminin) in serum-free mTeSR1 (STEMCELL Technologies) and plated at a density of 4 × 10^4^ cells cm^−2^ immediately prior to differentiation. When 40% confluency was reached, differentiation was initiated by replacing medium with endoderm differentiation medium: RPMI 1640 containing 1 × B27 (Life Technologies), 100 ng ml^−1^ Activin A (PeproTech) and 50 ng ml^−1^ Wnt3a (R&D Systems). Medium was changed every day for 3 days. On day 3, endoderm differentiation medium was replaced with hepatoblast differentiation medium: KO-DMEM (Life Technologies), Serum replacement (Life Technologies), 0.5% Glutamax (Life Technologies), 1% non-essential amino acids (Life Technologies), 0.2% β-mercaptoethanol (Life Technologies) and 1% DMSO (Sigma); and changed every second day for 5 days. On day 8, differentiating cells were cultured in the hepatocyte maturation medium HepatoZYME (Life Technologies) containing 1% Glutamax (Life Technologies), supplemented with 10 ng ml^−1^ hepatocyte growth factor (PeproTech) and 20 ng ml^−1^ oncostatin M (PeproTech). Media were renewed every second day for 12 days. Immunocytochemistry was performed as previously described [5]. Cytochrome P450 (CYP) activity was assessed. At day 25, hepatocytes were tested for CYP3A and CYP1A2 activity using P450-Glo technology (Promega) in accordance with the manufacturer's instructions. CYP activity was expressed as relative light units per millilitre and normalized by milligrams of protein (determined by BCA assay, Pierce).

### (e) Colony-forming unit cell assays

Haematopoietic differentiation of iPSCs and CFU-C assays was performed and scored as previously described [27].

### (f) iPSC cell-derived macrophage production

Adapted from previously published protocols [9,18], iPSC maintenance medium was changed in one confluent well of a six-well plate and replaced with 1.5 ml of StemPro hESC SFM (Gibco) supplemented with 50 ng ml^−1^ BMP4 (R&D), 50 ng ml^−1^ VEGF (R&D) and 20 ng ml^−1^ SCF (Life Technologies). Cells were passaged into two wells with 2.25 ml of fresh media using the EZPassageTM tool and embryoid bodies (EBs) were formed in suspension for 4 days (supplemented with cytokines on day 2). 10–15 EBs were transferred per well to a gelatin-coated six-well plate in X-VIVO15 media (3 ml per well) supplemented with 100 ng ml^−1^ CSF1 (BioLegend), 25 ng ml^−1^ IL3 (Peprotech), 2 mM Glutamax (Gibco), 0.05 mM β-mercaptoethanol (Gibco) and 1% v/v Penicillin-Streptomycin (Life Technologies) and media were replaced every three to four days for three weeks. Monocyte-like precursors were harvested from the supernatant (every three to four days for up to three months) and plated into untreated bacteriological plates or six-well plates in X-VIVO15 media supplemented with 100 ng ml^−1^ CSF1 (BioLegend), 2 mM Glutamax (Gibco) and 1% Penicillin-Streptomycin (Life Technologies) for 9–11 days.

### (g) Cytospins and rapid-chrome Kwik-Diff staining

Cells were harvested, re-suspended in PBS (2.5 × 10^4^ ml^−1^) and cyto-centrifuged at 72*g* for 7 min in a Thermo Shandon Cytospin 4 onto polylysine slides. After air-drying overnight, cells were stained with Kwik-Diff Staining according to the manufacturer's instructions (Thermo-Fisher)

### (h) Macrophage activation and plasticity

Adherent iPSC-derived macrophages were activated *in vitro* to three different phenotypes by treating them for 48 h with either 0.1 µg ml^−1^ LPS (Sigma) and 10 U ml^−1^ IFNγ (eBioscience), 20 ng ml^−1^ IL-4 (Preprotech) or 5 ng ml^−1^ IL-10 (Preprotech). In experiments designed to test macrophage plasticity, cells were activated in the first condition for 48 h then media was changed to the second condition for a further 48 h.

### (i) Quantitative real-time PCR

Total RNA was extracted using the RNAeasy Mini Kit (Qiagen); cDNA was generated from 500 ng or 1 µg of total RNA using the High Capacity cDNA synthesis Kit (Applied Biosystems). Two nanograms of cDNA was amplified per reaction and each reaction was performed in three technical replicates using the LightCycler 384 (Roche) with SYBR Green Master Mix II (Roche) and appropriate primers (electronic supplementary material, table S1). For gene expression analyses, *GAPDH*, *β-ACTIN* and *B2M* were used as reference genes and the geometrical mean was used to normalize the data.

### (j) Flow cytometry

Macrophages were detached using StemPro Accutase Cell Dissociation Reagent (Gibco), re-suspended and blocked with FcR Blocking Reagent (MACS) according to the manufacturer's instructions. 1 × 10^5^ cells were washed, stained with appropriate antibodies (electronic supplementary material, table S2) for 20 min at room temperature then washed within PBS with 1% BSA and 5 mM EDTA, then analysed using LSR Fortessa Analyser (BD) and FlowJo v. 10.2. Dead cells were gated out using DAPI.

### (k) Phagocytosis assay

Live imaging of phagocytosis was carried out as we described previously in [18] with some modifications to allow the use of pHrodo^TM^ Red Zymosan BioParticles for the analysis of SFCi55-ZsG macrophages. SFCi55-ZsG macrophages were stained with Hoechst33342 1 : 20 (Thermo-Fisher) for 20 min at 37 °C. A vial of pHrodo^TM^ Red Zymosan BioParticles was re-suspended in 2 ml of PBS and diluted 1 : 5. Imaging was performed on the Operetta High-Content Imaging System (Perkin-Elmer) at 5 min intervals for 175 min, at 40× magnification.

### (l) Statistical analyses

Results are expressed as mean ± s.e.m. Specific statistical tests (as stated in figure legends) were performed using Graph Pad software v. 6.0c. *p*-values less than 0.05 were considered statistically significant (*p < 0.05, **p < 0.01, ***p < 0.001, ****p < 0.0001).

## 3. Results

### (a) Production of SFCi55ZsG iPSC line

SFCi55 iPSCs were electroporated with the pZDonor-AAVS1-ZsGreen vector (figure 1*a*) and a plasmid expressing the AAVS1-specific zinc finger nuclease and selected in puromycin as previously described [24]. Genomic DNA isolated from puromycin-resistant clones was screened by PCR to identify clones in which the targeting vector had integrated into the *AAVS1* locus by homologous recombination. Over 90% of puromycin-resistant clones carried the correct targeting event (electronic supplementary material, figure S1A, B) and several clones were expanded and characterized. The specific clone, herein referred to as SFCi55ZsG, was shown to have a normal karyotype and retained the ability to generate cells of all three germ layers.

### (b) Expression of ZsGreen in undifferentiated iPSCs and in cells of all three germ layers

ZsGreen was expressed at high levels in all undifferentiated SFCi55-ZsGr iPSCs when grown in maintenance conditions (figure 1*b*). To assess whether the expression of ZsGreen persisted in terminally differentiated cells, we analysed the expression in EBs and in mature cells derived from all three germ layers. The SFCi55-ZsGr iPSCs line could be differentiated into cell types associated with all three germ layers. ZsGreen continues to be expressed at high levels in TUJ1-postiive neuronal cells (figure 1*c*) and in albumin-positive hepatocyte-like cells (figure 1*d*). CYP3A and CYP1A2 activity was detected in SFCi55-ZsG iPSCs-derived hepatocyte-like cells by luminescence (electronic supplementary material, figure S1C) and the level of activity of these cytochrome P450 enzymes was comparable to that observed for hepatocyte-like cells derived from other pluripotent stem cell lines (data not shown)

Differentiating SFCi55-ZsG iPSCs were cultured in semisolid CFU-C culture conditions to examine the haematopoietic colony-forming unit (CFU-C) derived from haematopoietic progenitors [27]. ZsGreen was expressed in a wide range of haematopoietic cell types generated under these conditions including cells of the erythroid, granulocytic and macrophage lineages (figure 1*e*). We then focused our study on the phenotypic and functional properties of macrophages.

### (c) SFCi55-ZsG-derived macrophages are comparable to control cells

**Figure 2.**
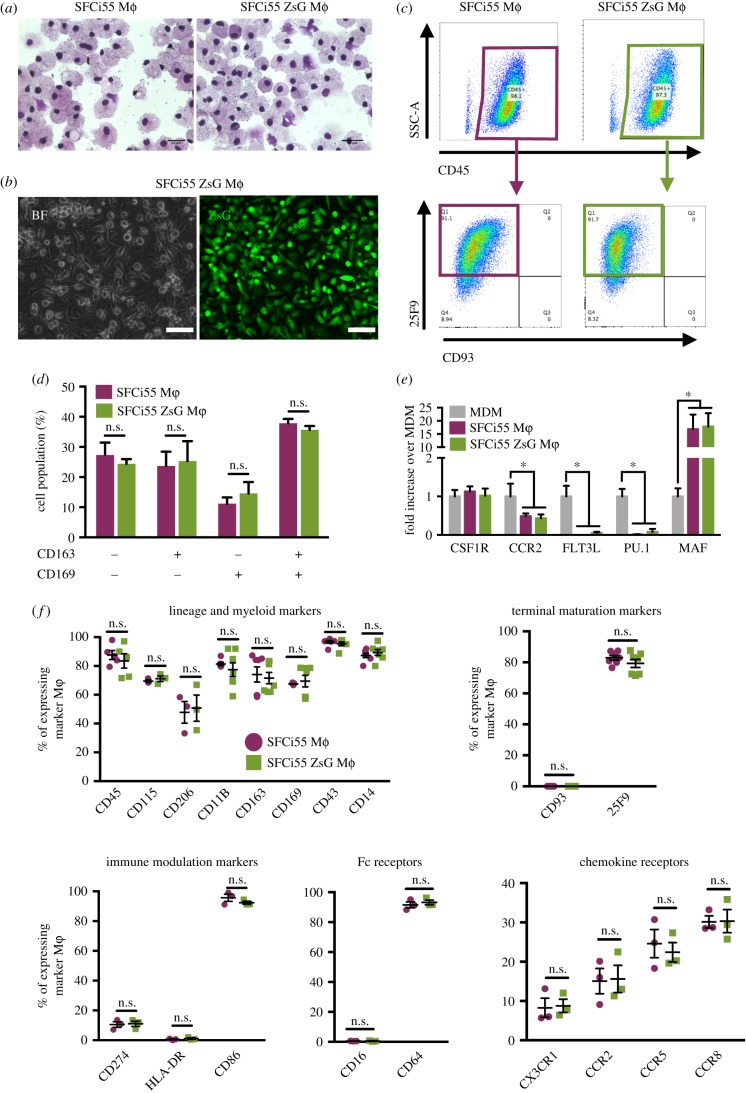
SFCi55-ZsG Macrophages are phenotypically comparable to control macrophages. (*a*) Cytospins of macrophages (Mϕ) derived from SFCi55 (parental line) and SFCi55-ZsG iPSCs (40×). (*b*) Bright field (BF) and green channel (ZsG) images of SFCi55-ZsG macrophages (40×). (*c*) Flow Cytometry analysis of CD45, CD93 and 25F9 expression in SFCi55 macrophages (left) and SFCi55-ZsG macrophages (right). (*d*) Quantification of flow cytometry analysis of CD163 and CD169 expression in SFCi55 and SFCi55-ZsG macrophages (*n* = 5, Mann–Whitney test). (*e*) Quantitation RT-PCR analyses of RNA isolated from monocyte-derived macrophages (MDM) and macrophages derived from the SFCi55 and SFCi55-ZsG iPSCs assessing the level of expression of genes associated with tissue resident macrophage genes (*MAF*) and monocyte-derived macrophages (*CCR2, FLT3, PU.1*). (*n* = 6, Kruskal–Wallis test with Dunn's multiple comparisons post-test) (*p < 0.05). (*f*) Quantification of flow cytometry analyses of cell-surface marker expression in macrophages derived from SFCi55 and SFCi55-ZsG iPSCs (*n* = 5 for all except CD115 and CD206; *n* = 3); maturation markers (*n* = 5), immune modulation markers (*n* = 3), Fc receptors (*n* = 3) and chemokine receptors (*n* = 3); Mann–Whitney test.

Macrophages generated from SFCi55-ZsG iPSCs had a similar morphology to macrophages derived from control SFCi55 iPSCs (figure 2*a*). Both lines generated approximately 5 × 10^6^ macrophages per six-well plate at each harvest indicating that the expression of ZsGreen had no significant effect on the efficiency of differentiation. All macrophages generated from the SFCi55-ZsGr iPSC line expressed ZsGreen at high levels (figure 2*b*; electronic supplementary material, figure S1D). The cell surface marker expression of monocyte-like and mature macrophages derived from SFCi55-ZsGr iPSCs was indistinguishable from those derived from the parental iPSC line (figure 2*c*; electronic supplementary material, figure S2A). When monocyte-like cells were first harvested, they expressed CD45 and CD93 but as they matured in culture, they gained expression of 25F9 and lost CD93 expression, as expected (figure 2*c*; electronic supplementary material, figure S2A). Expression of CD169 and CD163 was heterogeneous in macrophages derived from both cell lines (figure 2*d*; electronic supplementary material, figure S2B). We compared the expression of genes associated with monocyte/macrophage development and differentiation in iPSC-derived macrophages with monocyte-derived macrophages (MDM). The monocyte markers CCR2 and PU.1 are expressed at low levels in mature iPSC-derived macrophages from both lines compared with MDM (figure 2*e*). By contrast, a higher level of expression of MAF was observed in iPSC-derived cells, indicating that iPSC-derived macrophages are more comparable to tissue-resident macrophages [19] (figure 2*e*). We quantified the expression of specific cell surface markers associated with macrophage differentiation (CD45, CD115, CD206, CD11b, CD169, CD43 and CD14) and maturation (CD93, 25F9) as well as immune modulators (CD274, HLA-DR, CD86), Fc receptors (CD16, CD64) and chemokine receptors (CX3CR1, CCR2, CCR5, CCR8) on both SFCi55 and SCFi55–ZsG macrophages and observed a comparable proportion of cells expressing these markers (figure 2*f*) and a comparable level of marker expression per cell (electronic supplementary material, figure S3).

### (d) SFCi55-ZsG-derived macrophages are functional, can be activated and retain plasticity

Phagocytosis is one of the defining features of a functional macrophage and the rate of phagocytosis is closely associated with specific phenotypic states. We assessed phagocytosis in macrophages derived from SFCi55 and SFCi55-ZsG by live imaging using green and red Phrodo beads, respectively [18]. This strategy provides a quantitative measure of phagocytosis in real time and enables direct comparison between macrophages derived from different iPSC lines.

Comparable rates of phagocytosis were observed in naive macrophages derived from the SFCi55 and SFCi55-ZsG iPSC lines, indicating that the genetic manipulation had no functional consequences and that the sensitivity of the assay using the green and red Phrodo beads was comparable.

In response to environmental cues, macrophages have the ability to be activated to a specific functional state and to retain the ability to switch between these states [28,29]. To assess these features of iPSC-derived cells, macrophages derived from the SFCi55 and SFCi55-ZsG iPSCs were activated to three different phenotypes by treating with LPS and IFN*γ*, IL-4 and IL-10. These phenotypes are herein referred to as M(LPS + IFN*γ*), M(IL4) and M(IL10) [29].

**Figure 3.**
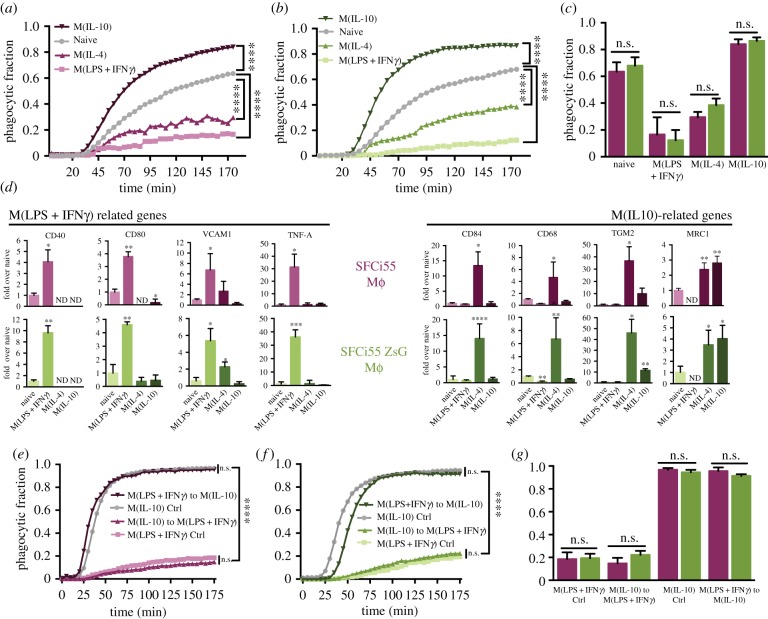
SFCi55-ZsG Macrophages are functional, can be activated and retain plasticity. (*a,b*) Fraction of phagocytic cells over time for SFCi55-derived (*a*) or SFCi55-ZsG-derived (*b*) macrophages (mϕ) in the naive state and activated using LPS + IFN*γ*, IL10 or IL4 (*n* = 6, two-way ANOVA and Dunnette's multiple comparisons post-test). (*c*) Quantification of phagocytic fractions (data from *a* and *b*) from SFCi55 and SFCi55-ZsG-derived macrophages in naïve and activated states (*n* = 6, Kruskal–Wallis test and Dunn's multiple comparisons post-test). (*d*) Quantitative RT-PCR of RNA isolated from naive and polarized macrophages derived from SFCi55 and SFCi55-ZsG macrophages to assess the expression of M(LPS + IFN*γ*) and M(IL10)-associated genes upon stimulation (*n* = 6, key to colours as in (*a*)). One-way ANOVA and Holm-Sidak's multiple comparisons post-test). (*e,f*) Fraction of phagocytic cells over time for SFCi55-ZsG macrophages (*e*) and SFCi55-ZsG macrophages (*f*) under M(LPS + IFN*γ*) and M(IL10) conditions only and where they were switched from M(LPS + IFN*γ*) to M(IL10) or M(IL10) to M(LPS + IFN*γ*) conditions (*n* = 6, two-way ANOVA and Tukey's multiple comparisons post-test). (*g*) Quantification of phagocytic fractions (*e*,*f* data) from SFCi55 and SFCi55-ZsG macrophages in experiments designed to assess plasticity (*n* = 6, Kruskal–Wallis test and Dunn's multiple comparisons post-test) (*p < 0.05, **p < 0.01, ***p < 0.001, ****p < 0.0001).

When macrophages were activated with LPS and IFN*γ* or IL-4, phagocytosis was significantly reduced compared with naive cells and this was evident in macrophages derived from both control and ZsGreen-expressing iPSCs (figure 3*a–c*; electronic supplementary material, figure S4). By contrast, when activated with IL10, a higher rate of phagocytosis was observed (figure 3*a–c*) and, again no significant difference in the response of SFCi55- and SFCi55ZsG-derived macrophages was observed (figure 3*a–c*). The activated phenotypes were confirmed by expression of genes encoding markers associated with M(LPS + IFN*γ*) macrophages (CD40, CD80, VCAM1, TNF-α) and M(IL10) or M(IL4) macrophages (CD84, CD68, TGM2, MRC1) and the expression levels of these genes were comparable between macrophages derived from the two iPSC lines (figure 3*d*). These data confirm that iPSC-derived macrophages can be activated effectively and that the high level of expression of ZsGreen in these macrophages had no detrimental effect on phagocytic function and on the ability of cells to be activated.

The plastic nature of macrophages is widely accepted, but this feature has not been tested directly in iPSC-derived macrophages. To assess whether iPSC-derived macrophages retained plasticity, they were first activated with IFN*γ* and LPS for 48 h then the medium was replaced with that containing IL10. The reciprocal experiment was also performed where IL10-activated macrophages were switched to M(IFN*γ* + LPS)-inducing conditions. Our quantitative phagocytosis assay demonstrates that LPS and IFNγ-induced iPSC-derived macrophages could be counter-activated to an M(IL10)-like phenotype and that the rate of phagocytosis was equivalent to macrophages that had been polarized with IL10 directly (figure 3*e,g*). Likewise, the rate of phagocytosis of macrophages that were first activated using IL10 then counter-activated with LPS and IFN*γ* was comparable to that of cells that had been activated with LPS and IFNγ only (figure 3*e,g*). Comparable data were obtained with macrophages derived from the SFCi55ZsG cell line, indicating that neither targeting of the *AAVS1* locus nor the high levels of ZsGreen expression affect the plasticity of iPSC-derived macrophages (figure 3*f,g*).

## 4. Discussion

Rapid advances over the past 10 years in the production of iPSCs and in the design of defined differentiation conditions have led to an enormous increase in the use of iPSC-derived cell types in drug testing and disease modelling [30]. Production and differentiation of patient-specific iPSCs has led to advances in the understanding and treatment of a wide range of genetic disorders. There has also been progress in the use of iPSC-derived cells in cell replacement therapies with recent reports describing the use of iPSC-derived retinal pigment epithelial cells in a few clinical case studies [31].

Several factors are inhibiting the clinical translation of iPSC-derived cell therapies including the significant safety issues concerning their genetic stability, the persistence of residual tumour-initiating iPSCs and the functional properties of the cells that are generated. The lack of appropriate animal models where therapeutic cells can be tracked, their efficacy monitored and their mechanism of action elucidated has further hampered progress [2,30]. As a first step to address some of these issues, we have tested a platform where transgenes can be inserted into a defined site in the iPSC genome and have created a genetically engineered iPSC line that expresses the ZsGreen fluorescent reporter. Targeting the reporter gene to the ‘safe harbour’, *AAVS1* locus, ensures that the reporter gene is not silenced and continues to be expressed in mature differentiated cells. This directed targeting strategy also ensures that the genetic manipulation could be reproduced in patient-specific iPSCs and importantly it reduces the risk of unwanted insertion mutagenesis that would be associated with random integration of transgenes into the genome.

The SFCi55-ZsG iPSC line can be differentiated into cells associated with all three primary germ layers and expression of the ZsGreen reporter persists in terminally differentiated TUJ1-expressing neurons, in albumin-expressing hepatocyte-like cells and in all cells of the haematopoietic lineage. ZsGreen-expressing hepatocytes had comparable cytochrome P450 activity to hepatocytes derived from other pluripotent stem cells, indicating that the genetic manipulation did not have a detrimental effect on cell function.

The therapeutic effect of macrophages has been demonstrated in animal models of fibrosis [12–14], but it has proved difficult to track these cells *in vivo* and there is a poor understanding of their mechanism of action. Translating the therapeutic effects of macrophages from animal models into human is becoming a real clinical need in different fields of investigation, such as cancer and inflammation. To date, the main strategies used to generate human macrophages *in vitro* have relied on pro- and monocytic cell lines such as U937, THP1 and Mono-mac cell lines [32–34] and monocyte-derived macrophages from peripheral blood monocytes [35,36]. Both systems are currently accepted and widely used in research, but they have significant limitations. Although cell lines are easy to culture, transfect and genetically modify, they are immortalized cells and thus may not fully represent primary human macrophages. Although MDM are likely to be more comparable to those found *in vivo,* the number of cells that can be generated is limited, there is significant variability between donors and they have proved difficult to genetically modify which is partly due to their expression of restriction factors that limit lentiviral integration [37]. Thus, the ability to generate functional macrophages from a limitless source of iPSCs that can be genetically manipulated represents a significant advance in the field.

A previous study reported the generation of macrophages from GFP-expressing iPSCs, but although this study demonstrated that GFP-expressing macrophages could be produced, no detailed functional comparisons were performed [22]. Our study demonstrates clearly that the macrophages produced from genetically engineered iPSCs are fully functional and indistinguishable from those generated from control iPSCs. We show that *AAVS1* targeting does not affect the production and function of iPSC-derived macrophages and, as the ZsGreen reporter is brighter than eGFP [38], we predict it to be a more sensitive strategy for *in vivo* tracking.

Macrophages can be activated to a whole spectrum of phenotypic states and can be modulated between these states depending on the microenvironment [28,29]. The local niche within a repairing and regenerating tissue is likely to be highly dynamic and the phenotype of transplanted ‘therapeutic’ cells will inevitably be influenced by that ever-changing environment. We demonstrate that iPSC-derived macrophages are capable of responding appropriately to environmental cues and can switch readily between phenotypes indicating that they could have a powerful therapeutic effect. The fact that they can be genetically manipulated also makes it possible to modulate their phenotype by genetic programming. The identification of programming factors and the stable expression of these using *AAVS1* targeting technology could lock macrophages into a desired phenotype and/or modulate how they respond to the regenerating environment *in vivo*.

## Supplementary Material

Supplementary Figures and Tables

## Data accessibility

This article has no additional data.
