# Supplementary material for: A human iPSC line capable of differentiating into functional macrophages expressing ZsGreen: a tool for the study and in vivo tracking of therapeutic cells
Source: Philos Trans R Soc Lond B Biol Sci. 2018 May 21;373(1750):20170219. doi: 10.1098/rstb.2017.0219 (PMC5974442; doi:10.1098/rstb.2017.0219)
Supplement: Supplementary Figures and Tables [file rstb20170219supp1.docx]

**Supplementary Figure Legends**

**Supplementary Figure S1. Production of SFCi55-ZsG iPSC line**

**A.** PCR Screening strategy to check for integration of targeting vector into the *AAVS1* locus; Primer 1 and 2 are complementary to sequences within the endogenous *AAVS1* locus (outside of homology arms), while primer 3 is complementary to the CAG promoter within the targeting vector.

**B.** Representative gel of PCR products using primers A and C (A) or B and C (B) generated from genomic DNA isolated from Puromycin-resistant iPSC colonies following targeting. Untargeted SFCi55 iPSC cells were used as a negative control.

**C**. SFCi55-ZsG hepatocytes’ metabolic activity measured in cytochromes CYP1A2 and CYP3A. Activity is expressed as relative light unit (RLU) per milliliter and normalised by milligram of protein.

**D.** Left: Representative flow cytometry histogram of macrophages (Mϕ) derived from control SFCi55 (purple) and SFCi55-ZsG (green) iPSCs for ZeissGreen expression, and right: quantification of mean fluorescent intensity (MFI) for ZeissGreen expression (n=6); Mann-Whitney Test.

**Supplementary Figure S2. Flow cytometry analysis of mature iPSC derived macrophages using antibodies to CD45, CD93, 25F9, CD163 and CD169.**

**A.** Representative flow cytometry dot-plots of SFCi55-derived monocyte precursors (left), macrophages (Mϕ) matured for 3 days and for 9 days demonstrating an increase in 25F9 expresion and loss of CD93 as macrophages mature. Functional studies were performed using fully mature 25F9^+^/CD93^-^ cells.

**B**. Representative flow cytometry dot-plots of macrophages (M) derived from control SFCi55 (left) and SFCi55-ZsG iPSCs (right) using antibodies to CD15, CD163 and CD169. No significant difference in marker expression was observed between macrophages derived from SFCi55 and SFCi55-ZsG iPSCs; macrophages are CD15^-^ and demonstrate heterogeneous expression of CD163 and CD169.

**Supplementary Figure S3. SFCi55 and SFCi55-ZsG macrophages are indistinguishable in terms of cell surface marker expression**

**A.** Representative flow cytometry histograms of macrophages (Mϕ) derived from control SFCi55 (purple) andSFCi55-ZsG (green) iPSCs, using antibodies to differentiation markers, maturation markers, immune modulation markers, Fc receptors and chemokine receptors (isotype controls (grey)).

**B.** Quantification of mean fluorescent intensity (MFI) of macrophages derived from SFCi55 (purple) and SFCi55-ZsG (green) iPSCs stained using antibodies to differentiation markers (n=5 for all except CD105 and CD206; n=3); maturation markers (n=5), immune modulation markers (n=3), Fc receptors (n=3) and chemokine receptors (n=3); Mann-Whitney Test. ΔMFI is sample MFI – MFI in Isotype control.

**Supplementary Figure S4.**

**A/B.** Representative mages of SFCi55-derived (A) or SFCi55-ZsG-derived (B) macrophages (Mϕ) in an naïve state or activated using IFNγ+LPS, IL4 or IL10.

**C/D.** Representative images of SFCi55-derived (C) or SFCi55-ZsG-derived (D) macrophages in ‘plasticity’ experiments where cells were either maintained in one activated state (IFNγ+LPS, IL10 controls) or switched from one state to another (IL10 to IFNγ+LPS or IFNγ+LPS to IL10).

[All images were acquired with a 40X objective at last time point: 175 min after appropriate beads (green or red) were added]
